# Implant-supported prosthetic rehabilitation after Ameloblastomas treatment: a systematic review

**DOI:** 10.1186/s12903-023-03765-7

**Published:** 2023-12-18

**Authors:** Mario Alberto Alarcón-Sánchez, Julieta Sarai Becerra-Ruíz, Lazar Yessayan, Seyed Ali Mosaddad, Artak Heboyan

**Affiliations:** 1 Biomedical Science, Faculty of Chemical-Biological Sciences, Autonomous University of Guerrero, Chilpancingo de los Bravo, Guerrero Mexico; 2 Institute of Research of Bioscience, University Center of Los Altos, University of Guadalajara, Tepatitlán de Morelos, Jalisco Mexico; 3 Department of Therapeutic Stomatology, Faculty of Stomatology, Yerevan State Medical University after Mkhitar Heratsi, Yerevan, Armenia; 4 Student Research Committee, School of Dentistry, Shiraz University of Medical Sciences, Qasr-e-Dasht Street, Shiraz, Iran; 5 Department of Prosthodontics, Faculty of Stomatology, Yerevan State Medical University after Mkhitar Heratsi, Str. Koryun 2, 0025 Yerevan, Armenia

**Keywords:** Dental implants, Survival rate, Dental prosthesis, Implant-supported, Ameloblastoma

## Abstract

**Background:**

Ameloblastoma (AM), the benign counterpart of ameloblastic carcinoma, is a benign odontogenic tumor of epithelial origin, naturally aggressive, with unlimited growth potential and a high tendency to relapse if not adequately removed. Patients with AM treated surgically can benefit from dental implant therapy, promoting oral rehabilitation and improving their quality of life. The present study aimed to determine the survival rate of dental implants placed after surgical treatment of patients affected by AM. In addition, there were two secondary objectives: 1) To evaluate which dental implant loading protocols are most frequently used and 2) To determine the type of prosthetic restoration most commonly used in these patients.

**Methods:**

The Preferred Reporting Items for Systematic Review and Meta-Analysis (PRISMA) guidelines were followed during the study. Searches were performed in three databases (PubMed/MEDLINE, Scopus, and Google Scholar) until November 2023. Additionally, the electronic search was enriched by an iterative hand search of journals related to oral pathology and medicine, maxillofacial surgery, and oral prosthodontics and implantology. Only reports and case series in English from January 2003 to date were included. The Joanna Briggs Institute tool (JBI-Case Reports/Case Series) was used for the study quality assessment.

**Results:**

The total number of patients and implants studied were 64 and 271, respectively, all with surgically treated AM. The patient’s ages ranged from 8 to 79 years, with a mean (SD) age of 37.3 ± 16.4. Fifty-three percent were male and 47% were female. The range of follow-up duration was 1 to 22 years. An implant survival/success rate of 98.1% was reported. In addition, most of them were conventionally loaded (38.3%). Hybrid implant-supported fixed dentures were the most commonly used by prosthodontists (53%).

**Conclusions:**

Oral rehabilitation with dental implants inserted in free flaps for orofacial reconstruction in surgically treated patients with AM can be considered a safe and successful treatment modality.

## Background

Ameloblastoma (AM) is a benign odontogenic tumor of epithelial origin, naturally aggressive, with unlimited growth potential and a high tendency to relapse if not adequately removed [1]. It represents 1% of tumors affecting the oral cavity and is the second most common benign neoplasm after odontoma, constituting approximately 9–11% of odontogenic tumors [2]. It shows a higher incidence between the fourth and fifth decade of life; however, it has no predilection for sex or race [3]. Regarding its location, 80% of all AM affects the posterior region of the mandible (corresponding to the site of the mandibular ramus, angle, and body), followed by the anterior part of the mandible and the posterior and anterior maxillary segments [4]. Nasal tract, orbital, and intracranial involvement are rare; however, they have been reported and can cause serious consequences [5–7]. AM are usually asymptomatic and, in most cases, are diagnosed in advanced stages. In this regard, the lesions that progress can reach a considerable size, generating erosion of bone tissue and invasion of neighboring tissues. Therefore, clinically, patients show swelling in the affected area, loss of teeth, malocclusion, airway obstruction, pathological fractures, and sometimes the tumor can become infected [8]. Radiographically, the classic pattern of AM is shown as a radiolucent, unilocular, or multilocular, well-defined lesion, which may show displacement of adjacent teeth and/or root resorption. Computed tomography is helpful for the evaluation of its extension and the degree of bone destruction [9].

According to the new classification of odontogenic tumors and maxillofacial lesions published by the World Health Organization (WHO) in 2022, five types are distinguished: conventional AM, formerly called solid/multicystic, unicystic (intraluminal, luminal, and mural), extraosseous/peripheral, metastatic and adenoid (recently introduced) [10]. Conventional AM is the most frequent and represents approximately 86–90% of all cases. According to its histologic findings, it can be classified into follicular, plexiform, acanthomatous, desmoplastic, basal, and granular. These patterns can occur individually or in combination; however, the follicular variant is the most frequent, followed by the plexiform variant [11]. The follicular pattern is characterized by the epithelial cells being arranged in islands or follicles surrounded by connective tissue, whereas, in the plexiform pattern, the epithelial cells are arranged in an interwoven plexiform network that outlines the adjacent connective tissue [3]. Thus, clinical features, imaging, and histopathological examination are needed together to confirm the definitive diagnosis of the lesion.

The management of AM remains a significant challenge for surgeons despite being one of the most common odontogenic neoplasms [12]. Some studies demonstrated a higher recurrence rate in patients after being treated by a conservative approach versus a radical one [13, 14]. Furthermore, a significant improvement in the quality of life of patients after surgical treatment of AM has been demonstrated [15]; therefore, the treatment of choice for this type of tumor remains surgical resection, which includes marginal or segmental resection with or without disarticulation depending on the extent of the tumor, together with periodic long-term follow-up (> 10 years). In addition, sufficient safety margins of 1.5 to 2 cm are recommended to prevent possible recurrence, followed by reconstruction of the resulting anatomical bone defect [16] using bone graft biomaterials such as autogenous grafts (derived from the same individual), which can be non-vascularized bone grafts or vascularized free flaps, allogenic (derived from another individual of the same species), xenogeneic (acquired from other species), alloplastic (commercially prepared) and customized (use of active biomolecules to regenerate bone) [17].

Patients with AM can benefit from dental implant therapy, which can be placed during surgery (primary placement) or after completion of surgical treatment (secondary placement), followed by implant-supported prosthetic rehabilitation using a fixed dental prosthesis, which can be cemented, screw-retained, or hybrid and/or a removable dental prosthesis [18]. In fact, it has been shown that patients with oral cancer mainly benefit from primary placement of dental implants for prosthetic rehabilitation with a 5-year survival rate of 92.8% compared to secondary placement (86.4%) [19], as well as a higher survival rate has been observed in those immediately [20] and delayed placed implants that had not received radiotherapy compared to previously irradiated sites [21]. On the other hand, a recent systematic review and meta-analysis reported an overall survival rate of 97% after 1 year of prosthetic loading following surgical resection of oral tumors and subsequent mandibular reconstruction with fibula free-flap. In this study, 69% of the tumors analyzed were benign and included AM. In addition, as part of the secondary objectives, the authors demonstrated a survival rate of 98% with immediate implants and 97% with delayed implants [22]. However, research on the survival rate of dental implants and subsequent implant-supported rehabilitation in surgically treated AM patients is scarce. Therefore, the present study aimed to determine the survival rate of dental implants placed subsequent to surgical treatment of patients affected by AM. In addition, two secondary objectives were set: 1) To evaluate which dental implant loading protocols are most frequently used and 2) To determine the type of prosthetic restoration most commonly used in these patients.

## Methods

### Protocol and methods

For the literature search and selection of studies, the present work was constructed following the Preferred Reporting Items for Systematic Review and Meta-Analysis (PRISMA) guidelines [23]. The protocol was not registered.

The electronic databases PubMed/MEDLINE, Scopus, and Google Scholar were consulted to investigate all available evidence on studies describing implant-supported prosthetic rehabilitation in surgically treated AM patients in detail. For this purpose, the Boolean terms “OR” and “AND” were used together with search header terms (MeSH). The PICO strategy was used, which consisted of Population (P)-patients affected by ameloblastoma, Intervention (I)-subjected to surgical treatment and subsequent implant-supported rehabilitation, Control (C)-not applicable, and Outcome (OR)-dental implant survival rate, loading protocol, type and functionality of prosthetic restorations used. Thus, the following research question was formulated: “What is the survival rate of dental implants placed after surgical treatment of patients affected by ameloblastoma?” with the following sub-questions: “What is the most frequent loading protocol?” and “What is the most common type of implant-supported prosthetic restoration used in these patients?”

### Eligibility criteria

The following characteristics were considered to select the best articles related to this research topic: On the one hand, articles from 2000 to date (2023) written in English were included. Regarding their design, only reports and case series were included. The articles included had to clearly present the confirmation of the histopathologic diagnosis of ameloblastoma, detail the type of intervention and reconstructive technique, the implant loading protocol, and the type of prosthetic restoration used, with a minimum follow-up of > 1 year. On the other hand, articles published before 2000 and those written in a language other than English were not considered. Cross-sectional clinical studies, cohort studies, narrative reviews, comprehensive reviews, systematic reviews, and meta-analyses were also excluded. Finally, studies showing insufficient data (lack of confirmatory tumor diagnosis, type of intervention, reconstructive technique, implant loading protocol, type of prosthetic restoration without follow-up or a follow-up < 1 year) were excluded from this study.

### Search strategy

The search was limited to case reports and case series only. A combination of keywords was used, including “Ameloblastoma,” “Dental implants,” “Fixed and removable dental prosthesis,” and/or “Implant-supported prosthetic rehabilitation.” The electronic search was enriched by an iterative hand search of journals related to oral pathology and medicine, maxillofacial surgery, and oral prosthodontics and implantology. The journals were the following: *“Journal of Oral Pathology & Medicine,” “Oral Surgery Oral Medicine Oral Pathology Oral Radiology,” “British Journal of Oral & Maxillofacial Surgery,” “Oral and Maxillofacial Surgery Clinics of North America,” Journal of Cranio-Maxillofacial Surgery“ and “Dentomaxillofacial Radiology,” Journal of Prosthodontics-Implant Esthetic and Reconstructive Dentistry*, *Journal of Prosthodontic Research*, *Journal of Advanced Prosthodontics*, *International Journal of Prosthodontics*, *European Journal of Prosthodontics and Restorative Dentistry*, *Journal of Esthetic and Restorative Dentistry*, *Journal of Prosthetic Dentistry*, *Journal of Indian Prosthodontics Society* and *Journal of Prosthodontics*. Table 1 shows the search strategy employed.

**Table 1:** The full search strategy used in the PubMed, Google Scholar, and Scopus databases

Database	Search Strategy
**PubMed**	(“Ameloblastoma”[Mesh]) AND “Dental Implants”[Mesh]) AND “Dental Prosthesis”[Mesh])
**Google Schoolar and Scopus**	TITLE-ABS-KEY (Ameloblastoma AND Dental Implants AND Dental Fixed OR Removable Prosthesis)

### Study selection

Initially, the studies were selected considering the articles’ titles and abstracts; any ambiguity in these sections was resolved by resorting to a full-text article. The articles found in the databases were subjected to a second review according to the eligibility criteria. If any conflict arose between the principal investigators (M.A.A.S and J.S.B.R), a third investigator (A.H) was consulted to resolve the debate.

### Quality assessment

Study quality was assessed following the guidelines (https://jbi.global/critical-appraisal-tools) in the individual case report and case series sections [24]. All included articles underwent quality assessment independently by two investigators (M.A.A.S and J.S.B.R). The instrument is based on a series of questions grouped according to the type of studies included in the systematic review that can be rated as: “Yes,” “No,” “Unclear” or “Not applicable.” According to the assessment tool, the risk of bias was classified as high when the study reached up to 49% of the “Yes” scores, moderate from 50 to 69%, and low when it reached above 70%.

### Data extractions and statistical analysis

From the previously selected articles, data extraction was performed by a third reviewer (A.H), and all relevant information such as The year of publication, first author, country, number of cases, study design, age, gender, characteristics of ameloblastoma such as histological type, location, clinical and imaging findings, type of surgical treatment, type of reconstruction, time after surgical reconstruction and the characteristics of the implant-supported prosthetic rehabilitation such as number of implants, the system used, position, size, loading protocol, complications, recurrence of MA after implant placement, survival rate, type of prosthesis, biomaterials used for the construction of the restorations and the follow-up period were extracted and recorded first in a standardized Excel data sheet and then in a database in the statistical program STATA V15. Finally, the selected articles were analyzed by descriptive statistics representing the data with mean ± standard deviation (SD), range (minimum-maximum), absolute and relative frequency. All the data were taken together to construct the systematic review.

## Results

### Selection of studies

**Fig. 1:**
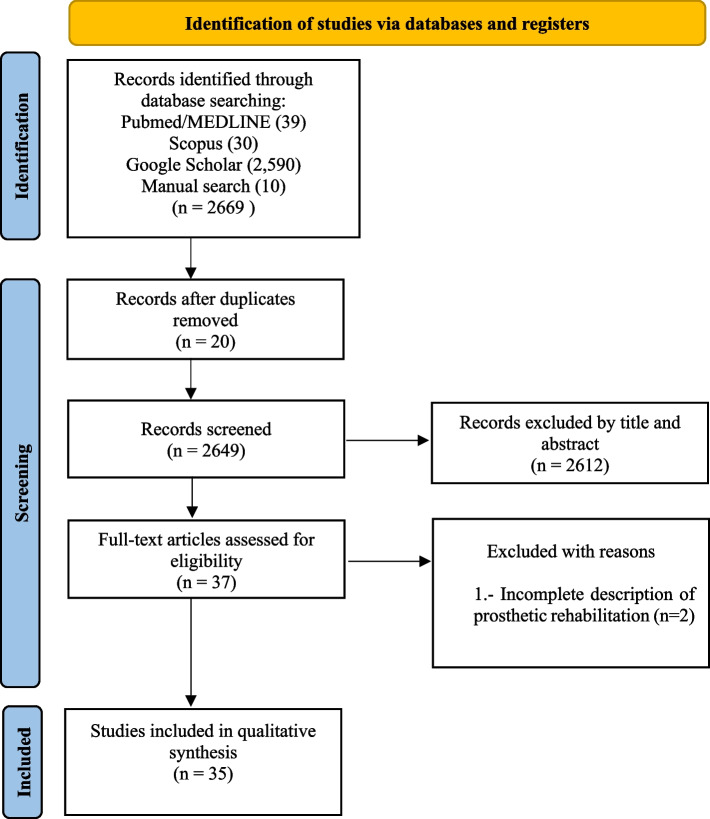
PRISMA flow diagram. PRISMA: Preferred Reporting Items for Systematic and Meta-Analyses

Initially, 2669 articles were found. Duplicates were eliminated, and based on the title and abstract, the remaining 2649 studies were reviewed. After analyzing the full text of the remaining articles, 2612 records were excluded as irrelevant. A total of 37 articles were assessed for eligibility, of which two studies were excluded because patient prosthetic rehabilitation was not fully described. Therefore, 35 articles were included in this systematic review (Fig. 1).

### Characteristics of the studies

Thirty-five investigations were reviewed in this study, of which 5 (14.3%) were case series [25–29] and 30 (85.7%) were case reports [30–59]. The total number of patients studied in the included investigations was 64, all with a history of surgically treated ameloblastomas and their subsequent implant-supported prosthetic rehabilitation. Most articles were published after 2013 (25: 71.4%). Four (24%) studies were conducted in India [42, 44, 55, 58], Turkey [27, 32, 47, 49] and Brazil [37, 46, 52], 3 (18%) in the USA [30, 35, 41], Spain [31, 50, 57], China [28, 33, 43], Korea [38, 39, 53], 2 (12%) in Italy [36, 48], Finland [29, 40] and other studies (6%) in Japan [54], Colombia [59], Iran [56], Taiwan [25], Austria [26] and Romania [45] (Tables 2 and 4).

**Table 2:** Clinicopathological characteristics of patients included in the systematic review

ID	Year/Author/Country	Cases No.	Study design	A/G	Ameloblastoma features				
					Histopathol.	Clinical-Location	Imaging	Treatment	Defect reconstruction/Time after reconstruction (Mo)
1	2003/Becker et al.*,*/USA [30]	1	Case Report	79/M	CVN	Asymptomatic Mandible P/L	RL, ML, WD, CE	RS-SM	Rib and tibial graft/NI
2	2004/Chana et al.*,*/Taiwan [25]	13	Case Series	5 = M 8 = F Mean age: 32 years	13 = CVN 5 = Recurrent	13 = Asymptomatic 13 = Mandible 7 = P, 3 = A, 3 = A + P, 4 = L, 6 = R	13 = RL, ML, CE	RS-SM	13 = Free fibula flap/NI
3	2007/Bueno et al.*,*/Spain [31]	1	Case Report	19/F	Unicystic	Asymptomatic Mandible P/L	RL, ML, WD, TD, RR, CE	RS-SM	Iliac bone graft 12
4	2007/Zemann et al.*,*/Austria [26]	7	Case Series	4 = M 3 = F Mean age: 49	6 = CVN 1 = Unicystic 1 = Folicular, 4 = Plexiform, 1 = Acanthomatous	7 = Asymptomatic 5 = Mandible 2 = Maxilla 3 = P, 1 = A, 3 = A + P 4 = L, 2 = R	7 = RL, 5 = UL, 2 = ML, 7 = WD 7 = CE	5 = RS-SM 2 = RS-HMX	2 = Scapule bone graft 5 = Iliac bone graft 6
5	2008/ Kürkcü et al.*,*/Turkey [32]	2	Case Report	42/F 41/F	2 = CVN	2 = Asymptomatic 2 = Mandible P/R	2 = RL, ML,WD,RR,CE	2 = RS-SM	2 = Free fibula flap + DO 16.5
6	2010/Wong et al.*,*/China [33]	1	Case Report	66/M	CVN	Asymptomatic Mandible A + P/L	RL, ML, WD, CE	RS-MM	Iliac bone graft 24
7	2011/Paranque et al.*,*/France [34]	1	Case Report	49/M	CVN Folicular	Asymptomatic Mandible A + P/L	RL, ML, WD, TD, CE	RM-SM	Free fibula flap 3
8	2011/Tözüm et al.*,*/Turkey [27]	3	Case Series	2 = M 1 = F Mean age: 36.3	3 = CVN	3 = Asymptomatic 3 = Mandible 3 = P, 1 = L, 2 = R	3 = RL, ML, WD, TD, RR, CE	3 = RS-HM	3 = Iliac bone graft 6
9	2011/Minichetti et al.*,*/USA [35]	1	Case Report	25/M	CVN	Asymptomatic Mandible-A	RL, ML, WD, TD, RR, CE	RS + SM	Iliac bone graft 5
10	2012/Oteri et al.*,*/Italy [36]	1	Case Report	66/M	CVN	Asymptomatic Mandible P/R	RL, ML, WD, TD, RR, CE	RS + SM	Free fibular flap + DO NI
11	2013/Pereira et al.*,*/Brazil [37]	1	Case Report	26/M	CVN Plexiform	Asymptomatic Mandible A + P/L	RL,ML,WD,TD,RR,CE	RS + SM	Iliac bone graft 6
12	2013/Kim et al.*,*/Korea [38]	2	Case Report	19/F 21/F	2 = CVN Follicular	2 = Asymptomatic 2 = Mandible P/R	2 = RL, ML, WD, TD, RR, CE	2 = RS + SM	2 = Iliac bone graft 12
13	2013/Kim et al.*,*/Korea [39]	1	Case Report	29/M	CVN Plexiform	Asymptomatic Mandible A + P/R	RL, ML, WD, TD, RR, CE	RS-SM	Block bone graft Stem cells
14	2013/Sándor et al.*,*/Finland [40]	1	Case Report	55/M	CVN	Asymptomatic Mandible A	RL, ML, WD, TD, RR, CE	RS-SM	Stem cells
15	2013/Cheung et al.*,*/China [28]	4	Case Series	1 = M 3 = F Mean age: 40	4 = CVN 1 = Recurrent	Asymptomatic 4 = Mandible 1 = P, 2 = A, 1 = A + P	4 = RL, ML, WD, CE	4 = RS-SM	4 = Free fibula flap + DO 23.8
16	2013/Wolf et al.*,*/Finland [29]	3	Case Series	2 = M 1 = F Mean age: 49.3	3 = CVN 3 = Recurrent	Asymptomatic 3 = Mandible 2 = P, 1 = A, 2 = L	3 = RL, ML, WD, TD, RR, CE	3 = RS-SM	3 = Stem cells 12
17	2013/Levine et al.*,*/USA [41]	2	Case Report	28/F 20/M	2 = CVN	Asymptomatic 2 = Mandible A + P/L, P/R	2 = RL, ML, WD, TD, RR, CE	2 = RS-SM	2 = Free fibula flap with dental implants
18	2013/Natashekara et al./India [42]	1	Case Report	56/M	Unicystic	Asymptomatic Mandible A	RL, ML, WD, TD, RR	RS-SM	Iliac bone graft 3
19	2013/Wang et al.*,*/China [43]	1	Case Report	56/M	CVN	Asymptomatic Mandible P/L	RL, ML, WD	RS-SM	Rib bone graft 96
20	2013/Christian et al.*,* /India [44]	1	Case Report	23/F	CVN	Asymptomatic Mandible P/R	RL, ML, CE	RS-SM	Healing obturator 6
21	2014/Cioranu et al.*,*/Romania [45]	1	Case Report	8/M	CVN Folicular	Asymptomatic Mandible P/R	RL, ML, WD, TD, RR, CE	RS-SM	Iliac bone graft 11
22	2015/Lustosa et al.*,*/Brazil [46]	1	Case Report	44/M	CVN Follicular	Asymptomatic Mandible P/R	RL, ML, WD, TD, RR, CE	RS-SM	rhBMP-2 + bovine bone graft 6
23	2015/Askin et al.*,* /Turkey [47]	1	Case Report	29/F	CVN	Asymptomatic Mandible P/R	RL, ML, WD, TD, RR, CE	RS-SM	Iliac bone graft 6
24	2017/Bucci and Nocini,/Italy [48]	1	Case Report	53/M	CVN Recurrent	Asymptomatic Mandible P/R	RL, ML, WD, TD, RR	RS-MM	Fresh frozen bone- allogenic 9
25	2017/Ozen et al.*,* Turkey [49]	1	Case Report	20/F	CVN Plexiform	Asymptomatic Mandible P/R	RL, ML, WD, TD, RR, CE	RS-SM	Iliac bone graft NI
26	2017/Sanz-Alonso et al.*,*/Spain [50]	1	Case Report	38/M	Unicystic Recurrent	Asymptomatic Maxilla P/L	RL, ML, WD, TD, RR, CE	RS-SM	Block bone graft 6
27	2019/Toure and Gouet,/France [51]	1	Case Report	50/M	CVN Recurrent	Asymptomatic Mandible A + P/R	RL, ML, WD, TD, RR, CE	RS-HM	3D custom-made porous titanium plate/Same day
28	2019/Ribeiro-Junior et al.*,*/Brazil [52]	1	Case Report	32/F	CVN Desmoplastic	Symptomatic Mandible A	RL, ML, WD, TD, RR, CE	RS-MM	Titanium plate and screws-2.4 system/Same day
29	2019/Lee et al.*,*/Korea [53]	1	Case Report	28/M	CVN	Asymptomatic Mandible A + P/R	RL, ML, WD, TD, RR	RS-MM	DO 48
30	2020/Ishidara et al.*,*/Japan [54]	1	Case Report	20/F	CVN	Asymptomatic Mandible A/	NI	RS-MM	DO 144
31	2020/Nag and Bhagwatkar,/India [55]	1	Case Report	36/M	CVN	Symptomatic Mandible P/L	RL, WD, CE	RS-HM	Free fibula flap 72
32	2021/Niakan and Yaghoobi,/Iran [56]	2	Case Report	14/M 22/F	2 = CVN	2 = Asymptomatic 2 = Mandible 2 = P/L	2 = RL, ML, WD, TD, RR, CE	2 = RS-HM	2 = Iliac bone graft NI
33	2021/Garrido-Martínez et al.*,*/Spain [57]	1	Case Report	61/F	CVN	Asymptomatic Maxilla P/L	CT showed a lobulated polyp of approximately 3 cm	RS-HMX	Free fibula flap/Same day
34	2022/Srivastava et al.*,*/India [58]	1	Case Report	31/M	CVN	Asymptomatic Mandible A + P/L	RL, ML, WD	RS-SM	Free fibula flap 18
35	2022/Ardila et al.*,*/Colombia [59]	1	Case Report	42/F	CVN	Asymptomatic Mandible P/L	RL, ML, WD, RR	RS-SM	3D custom-made porous titanium plate NI

*Abbreviations:* *NI* No information, *F* Female, *M* Male, *CVN* Conventional, *A* Anterior, *P* Posterior, *L* Left, *R* Right, *CE* Cortical expansion, *RL* Radiolucent, *ML* Multilocular, *UL* Unilocular, *WD* Well-defined, *TD* Tooth displacement, *RR* Root resorption, *RS* Resection surgical, *MRZ* Marsupialization, *CTG* Curettage, *MM* Marginal mandibulectomy, *SM* Segmental mandibulectomy, *HM* Hemimandibulectomy, *HMX* Hemimaxilectomy, *Mo*. Months

### Clinicopathologic characteristics of the patients

The ages of the patients ranged from 8 to 79 years; the mean ± (SD) age of the patients studied was 37.3 ± 16.4 years, of whom 53% were male and 47% were female. A total of 12 (19%) cases were reported as recurrent AM, which were reintervened. It was also observed that 94% of patients had conventional/multicystic AM [25, 27–30, 32–49, 51–59], and only 4 (6%) cases were diagnosed as unicystic type AM [26, 31, 42, 50]. The most common histologic variant was the plexiform type (11%) [26, 37, 39, 49], followed by the follicular variant (8%) [26, 34, 38, 45, 46]. The most affected anatomical region was the mandible (94%) [25, 27–49, 51–56, 58, 59], mainly the posterior (58.3%) and left side of the face (42%), followed by the posterior maxilla (75%) [26, 50, 57]. Clinically, 97% of patients were asymptomatic (did not report pain) [25–51, 53, 54, 57–59], and 91% presented cortical expansion [25–41, 44–47, 49–52, 55, 56, 59]. Radiographically, 97% of the lesions were radiolucent [25–53, 55, 56, 58, 59], multilocular (89.10%) [25, 27–53, 56, 58, 59], well-defined (73.43%) [26–43, 45–53, 56, 58, 59]. Bone resorption was present in 50% [27, 29, 31, 32, 35–43, 45–53, 56] of the cases, and 45.31% of the cases had a displacement of teeth adjacent to the tumor lesion [27, 29, 31, 32, 35–42, 56]. The treatment of choice was surgical resection of the tumor (100%) [25–59], with segmental mandibulectomy being the most frequent procedure (77%) [25, 28–47, 49, 50]. For reconstruction of the resulting bone defect, 41% of the cases were reconstructed by fibula free-flap [25, 28, 32, 34, 36, 41, 55, 57, 58], followed by iliac bone graft (31.2%) [26, 27, 31, 33, 35, 37, 38, 42, 45, 47, 49, 56]. Finally, the mean time after maxillofacial reconstruction for dental implant placement was 23.00 ± 34.00 months, equivalent to 1.9 years (Tables 2 and 4).

### Characteristics of the implant-supported prosthetic rehabilitation of patients

A total of 261 implants were studied in the included investigations, of which only 4 (2%) [29, 40, 53, 56] failed. In addition, no relapses of the tumor lesion after dental implant placement were reported. The survival rate was 98.1%, and the range of follow-up duration was 1–22 years. After implant placement, 38.3% were conventionally loaded [28, 30, 31, 33, 36–40, 43, 45, 48, 54, 56, 58], followed by the immediate loading protocol (28%) [25, 34, 35, 41, 42, 52]. In total, 62 implant-supported restorations were placed, of which 79% corresponded to fixed dentures [25, 28, 29, 31–36, 38, 39, 41–44, 46–52, 54–59] and 21% to removable dentures [26, 27, 30, 33, 37, 40, 45, 53]. Regarding fixed prostheses, the most used types were hybrid prostheses (53%) [43, 49, 52, 56–59], followed by cemented restorations (27%) [35, 42, 44, 55]. Of the removable prosthesis, metal bar retention was the most frequently used anchorage system (86%) [26, 27, 30, 33, 37, 55]. The most commonly used biomaterials for the construction of prosthetic devices were metal-ceramic (55%) [34, 35, 42, 44–46] for the fixed systems and acrylic resin (27.3%) for the removable systems [30, 33, 55] (Tables 3 and 4).

**Table 3:** Characteristics of implant-supported rehabilitation of patients after ameloblastoma treatment

ID	Author	Oral rehabilitation features										
		No.	System	Position	Size	Loading protocol	Complications		Relapse of AM	Survival rate (%)	Implant-supported prosthesis/Biomaterial	Follow-up (years)
							Failure	Type				
1	Becker et al.*,* [30]	5	Br*å*nemark®(Nobel Biocare™)	#31,32,41,42	NI	5 = CVN	No	No	No	100	Removable, metallic bar-retained/Acrylic resin	2.5
2	Chana et al.*,* [25]	44	3I System, Implant, Innovations, Inc.	NI	NI	44 = IMT	No	1 = Bone loss	No	100	13 = Fixed	5
3	Bueno et al.*,* [31]	4	Br*å*nemark®(Nobel Bicare™)	#33–36	NI	4 = CVN	No	No	No	100	Fixed	10
4	Zemann et al.*,* [26]	42	NI	NI	NI	NI	No	No	No	100	5 = Removable, metallic bar-retained 2 = Fixed	11.5
5	Kürkcü et al.*,* [32]	6	Straumann® Euroteknika®	#44–46 #44–46	4.1 × 12 = 6	6 = E	No	No	No	100	1 = Fixed NI	2
6	Wong et al.*,* [33]	4	Br***å***nemark®(Nobel Bicare™)	#32,33,41,43	4 × 18 = 4	4 = CVN	No	No	No	100	Removable, metallic bar and magnet-retained/Acrylic resin	3
7	Paranque et al.*,* [34]	6	OsseoSpeed, Astra Tech AB	#31–36	4 × 13 = 2 4 × 15 = 4	6 = IMT	No	No	No	100	Fixed, screw-retained/Metal-ceramic	5.5
8	Tözüm et al.*,* [27]	9	NI	#43,45 #34–37 #35–37	4.8 × 14 = 8 4.8 × 10 = 1	9 = CVN	No	No	No	100	1 = Removable, metallic bar-retained 2 = Fixed	1
9	Minichetti et al.*,* [35]	6	ZimVie®	#32–35,42,43	3.75 × 10 = 3 3.75 × 14 = 3	6 = IMT	No	No	No	100	Fixed, cement-retained/Metal-ceramic	8
10	Oteri et al.*,* [36]	7	Phibo®	#33,36,37,41,42,46,47	3.3 × 8.5 = 2 4.1 × 8.5 = 1 4.3 × 8.5 = 1 4.7 × 8.5 = 1 4.7 × 11.5 = 2	7 = CVN	No	No	No	100	Fixed, screw-retained	2
11	Pereira et al.*,* [37]	4	Straumann™ Neodent®	#33,36,43,46	5 × 15 = 4	4 = CVN	No	No	No	100	Removable, metallic bar-retained	5
12	Kim et al.*,* [38]	4 2	Implantium™ Implantium™	#44–47 #34,36	NI NI	6 = CVN	No No	No No	No No	100	2 = Fixed	6
13	Kim et al.*,* [39]	5	Xive® Dentsply Sirona™	#31,42,43,44,46	NI	5 = CVN	No	No	No	100	Fixed	3
14	Sándor et al. [40]	6	NI	#31,33,34,41,44	NI	6 = CVN	Yes/1	No	No	83.3	Removable	3
15	Cheung et al.*,* [28]	18	Br***å***nemark®(Nobel Bicare™)	#32,34,35,42,44,45 #31,33,42,44,46 #33,41,43,45 #44–46	4 × 13 = 16 4 × 11.4 = 1 4 × 11.5 = 1	18 = CVN	No	1 = Bone loss	No	100	4 = Fixed	2.4
16	Wolf et al.*,* [29]	6 1	Straumann SLActive Straumann AG	#31,32,35 #36	NI	7 = CVN	Yes/1 No	No	No	83.3 100	1 = Fixed NI	3.5 1.7
17	Levine et al.*,* [41]	6 4	NI	#33–36,41,43 NI	NI	10 = IMT	No No	No Pneumonia	No No	100	2 = Fixed	1.8 1
18	Natashekara et al. [42]	2	HI-TEC®	#31,41	4.2 × 10 = 2	2 = IMT	No	No	No	100	Fixed, cement-retained/Metal-ceramic	2
19	Wang et al.*,* [43]	3	Straumann®	#33,34,36	NI	3 = E	No	No	No	100	Fixed, hybrid /Metal-ceramic	3
20	Christian et al.*,* [44]	2	Zimmer Biomet®	#45,46	4.7 × 11 = 2	2 = E	No	No	No	100	Fixed, cement-retained/Metal-ceramic	2
21	Cioranu et al.*,* [45]	4	NI	#32,42,43,45	NI	4 = CVN	No	No	No	100	Removable	22
22	Lustosa et al.*,* [46]	4	Straumann®	#43,44,45,46	NI	4 = E	No	No	No	100	Fixed	5
23	Askin et al.*,* [47]	7	Swiss Plus®	#32,33,42-44,46,47	3.7 × 14 = 4 4.8 × 14 = 3	7 = CVN	No	No	No	100	Fixed	6
24	Bucci and Nocini, [48]	3	Br***å***nemark®(Nobel Bicare™)	#43–45	4 × 8.5 = 2 4 × 13 = 1	3 = CVN	No	No	No	100	Fixed	1
25	Ozen et al.*,* [49]	2	BioHorizons®	#43,46	3.8 × 10.5 = 1 4.6 × 10.5 = 1	2 = E	No	No	No	100	Fixed, hybrid/Ceramic	2
26	Sanz-Alonso et al.*,* [50]	2	Normon Dental®	#24,25	3.75 × 11.5 = 2	NI	No	No	No	100	Fixed	7
27	Toure and Gouet, [51]	2	Materialise®	#44,45	NI	2 = E	No	No	No	100	Fixed, screw-retained/Metal	1
28	Ribeiro-Junior et al.*,* [52]	5	Neodent®	#31,33,41,43,45	3.5 × 7 = 3 3.5 × 8 = 1 3.5 × 9 = 1	5 = IMT	Yes/1	No	No	83.3	Fixed, hybrid	6
29	Lee et al.*,* [53]	6	Hiossen®	#31,41,43–46	NI	6 = E	No	No	No	100	Removable, metallic bar-retained/Acrylic resin	3
30	Ishidara et al.*,* [54]	3	NI	#31,33,41	NI	3 = CVN	No	No	No	100	Fixed	3
31	Nag and Bhagwatkar, [55]	4	Bioline Dental®	#31,33,34,36	3.7 × 13 = 2 3.7 × 16 = 2	4 = E	No	No	No	100	Fixed, cement-retained/Metal-ceramic	3
32	Niakan and Yaghoobi, [56]	4 3	Implantium, Dentium® Straumann®	#33–35 #34–36	3.8 × 12 = 4 4.1 × 10 = 3	4 = E 3 = CVN	Yes/1	No	No	85.7	2 = Fixed, hybrid 1 = Metal-ceramic	3
33	Garrido-Martínez et al.*,* [57]	8	Zimmer Biomet®	#13,16-18,23,24,27,28	4 × 10 = 8	8 = E	No	No	No	100	Fixed, hybrid	4
34	Srivastava et al.*,* [58]	5	Hiossen®	#32,33, 36,41,44	10 × 4 = 3 10 × 3.5 = 2	5 = CVN	No	No	No	100	Fixed, hybrid	4
35	Ardila et al.*,* [59]	3	NI	#44–46	4,1 × 10 = 3	3 = E	No	No	No	100	Fixed, hybrid	4

*Abbreviations:* *NI* No information, *CVN* Conventional, *IMT* Immediate, *E* Early, *AM* Ameloblastoma. The FDI Dental Numbering System was used as a reference to name the position of the dental implants

**Table 4:** Summary of variables included in the study

Parameters	Values	%
**Articles**	35	
**Total Cases**	64	
**Age (years)**		
Mean ± SD	37.3 ± 16.4	
Range (Min-Max)	8–79	
**Gender**		
Male	34	53.00
Female	30	47.00
**Ameloblastoma Type**		
Conventional	60	94.00
Unicystic	4	6.00
Recurrent	12	19.00
**Histological Variant**		
Follicular	5	8.00
Plexiform	7	11.00
Acanthomatous	1	2.00
Desmoplastic	1	2.00
**Jaws**		
**Maxilla Region**	4	6.00
Anterior	–	–
Posterior	3	75.00
Anterior+Posterior	1	15.00
Left	2	50.00
Right	2	50.00
**Mandible Region**	60	94.00
Anterior	12	20.00
Posterior	35	58.33
Anterior+Posterior	13	22.00
Left	25	42.00
Right	22	37.00
**Clinical**		
Symptomatic	2	3.12
Asymptomatic	62	97.00
Cortical Expansion	58	91.00
**Imaging**		
Radiolucent	62	97.00
Unilocular	6	9.40
Multilocular	57	89.10
Well-Defined	47	73.43
Tooth Displacement	29	45.31
Root Resorption	32	50.00
**Surgical Treatment**		
Resection Surgical	64	100
Marginal Mandibulectomy	6	9.40
Segmental Mandibulectomy	49	77.00
Hemimandibulectomy	6	9.40
Hemimaxilectomy	3	4.70
**Defect Reconstruction**		
Rib Bone Graft	2	3.12
Tibial Bone Graft	1	2.00
Free Fibula Flap	26	41.00
Iliac Bone Graft	20	31.20
Scapula Bone Graft	2	3.12
rhBMP-2 + bovine bone graft	1	2.00
Fresh frozen bone- allogenic	1	2.00
Block Bone Graft	3	4.70
Titanium plate and screws-2.4 system	1	2.00
Healing obturator	1	2.00
**Defect Reconstruction**		
Distraction Osteogenesis	10	16.00
3D custom-made porous titanium plate	2	3.12
Stem cells	5	8.00
**Time after reconstruction for placement of dental implants (*Months*)**		
Mean ± SD	23.00 ± 34.00	
**Dental implant features:**		
**Implants (*number*)**	261	
**Loading protocol**		
-Immediate	73	28.00
-Early	44	17.00
-Conventional	100	38.31
**Failures**		
-Yes	4	2.00
-No	257	98.40
**Ameloblastoma recurrence after dental implant placement**		
-Yes		
-No	–	–
**Survival rate (%)**	64	100.00
Mean ± SD	98.1 ± 5.3	
**Prosthetic features:**		
**Prosthesis (*number*)**	62	
**Type**		
Removable	13	21.00
Fixed	13	79.00
**Anchorage system**		
**Removable**		
Metallic bar-retained	6	86.00
Magnet-retained	1	14.00
**Fixed**		
Screw-retained	3	20.00
Cemented-retained	4	27.00
Hybrid	8	53.00
**Prosthetic biomaterial**		
Acrylic Resin	3	27.30
Metal	1	9.10
Metal-ceramic	6	55.00
Ceramic free metal	1	9.10
**Follo-up (years)**		
Mean ± SD	4.31 ± 3.82	
Range (Min-Max) (years)	1–22	

Data were reported with mean ± standard deviation and *n* (%)

### Quality evaluation

Tables 5 and 6 show the results of the quality assessment of the included studies. Based on the checklist used to score the articles, all studies achieved scores > 70 [25–59], resulting in a low risk of bias in all selected studies.

**Table 5:** Results of the quality assessment for included studies

Authors	Q1	Q2	Q3	Q4	Q5	Q6	Q7	Q8	Overall score and quality
**Becker et al.*,*** [30]	Y	Y	Y	Y	Y	U	Y	Y	87.5
**Bueno et al.*,*** [31]	Y	U	Y	Y	Y	Y	Y	Y	87.5
**Kürkcü et al.*,*** [32]	Y	Y	Y	Y	U	Y	Y	Y	87.5
**Wong et al.*,*** [33]	Y	Y	Y	Y	Y	Y	Y	Y	100
**Paranque et al.*,*** [34]	Y	Y	Y	Y	Y	Y	Y	Y	100
**Minichetti et al.*,*** [35]	Y	Y	Y	Y	Y	Y	Y	Y	100
**Oteri** ***et sal.,*** [36]	Y	Y	Y	Y	Y	Y	Y	Y	100
**Pereira et al.*,*** [37]	Y	Y	Y	Y	Y	Y	Y	Y	100
**Kim et al.*,*** [38]	Y	Y	Y	Y	Y	Y	Y	Y	100
**Kim et al.*,*** [39]	Y	Y	Y	Y	Y	Y	Y	Y	100
**Sándor et al.*,*** [40]	Y	Y	Y	Y	Y	Y	Y	Y	100
**Levine et al.*,*** [41]	Y	U	Y	U	Y	Y	Y	Y	75
**Natashekara et al.** [42]	Y	Y	Y	Y	Y	Y	Y	Y	100
**Wang et al.*,*** [43]	Y	Y	Y	Y	Y	Y	Y	Y	100
**Christian et al.*,*** [44]	Y	Y	Y	Y	Y	Y	Y	Y	100
**Cioranu et al.*,*** [45]	Y	Y	Y	Y	Y	Y	Y	Y	100
**Lustosa et al.*,*** [46]	Y	Y	Y	Y	Y	Y	Y	Y	100
**Askin et al.*,*** [47]	Y	Y	Y	Y	Y	Y	Y	Y	100
**Bucci and Nocini,** [48]	Y	Y	Y	U	Y	Y	Y	Y	87.5
**Ozen et al.*,*** [49]	Y	Y	Y	Y	Y	Y	Y	Y	100
**Sanz-Alonso et al.*,*** [50]	Y	Y	Y	Y	Y	Y	Y	Y	100
**Toure and Gouet,** [51]	Y	Y	Y	Y	Y	Y	Y	Y	100
**Ribeiro-Junior et al.*,*** [52]	Y	Y	Y	Y	Y	Y	Y	Y	100
**Lee et al.*,*** [53]	Y	Y	Y	Y	Y	Y	Y	Y	100
**Ishidara et al.*,*** [54]	Y	Y	Y	Y	Y	Y	Y	Y	100
**Nag and Bhagwatkar,** [55]	Y	Y	Y	Y	Y	Y	Y	Y	100
**Niakan and Yaghoobi,** [56]	Y	Y	Y	Y	Y	Y	Y	Y	100
**Garrido-Martínez et al.*,*** [57]	Y	Y	Y	Y	Y	Y	Y	Y	100
**Srivastava et al.*,*** [58]	Y	Y	Y	Y	Y	Y	Y	Y	100
**Ardila et al.*,*** [59]	Y	Y	Y	Y	Y	Y	Y	Y	100

Question (Q); N/A, not aplicable; Y, yes; U, unclear

Q1: Were patient’s demographic characteristics clearly described?

Q2: Was the patient’s history clearly described and presented as a time line?

Q3: Was the current clinical condition of the patient on presentation clearly described?

Q4: Were diagnostic tests or assessment methods and the results clearly described?

Q5: Was the interventio(s) or treatment procedure (s) clearly described?

Q6: Was the post-intervention clinical condition clearly described?

Q7: Were adverse events (harms) or unanticiped events identified and described?

Q8: Does the case report provide takeway lessons?

**Table 6:** Results of the quality assessment for included studies

Authors	Q1	Q2	Q3	Q4	Q5	Q6	Q7	Q8	Q9	Q10	Overall score and quality
**Chana et al.*,*** [25]	Y	Y	Y	Y	Y	Y	Y	Y	N	Y	90
**Zemann et al.*,*** [26]	Y	Y	Y	Y	Y	Y	Y	Y	Y	Y	100
**Tözüm et al.*,*** [27]	Y	Y	Y	Y	Y	Y	Y	Y	Y	Y	100
**Cheung et al.*,*** [28]	Y	Y	Y	Y	Y	Y	Y	Y	Y	Y	100
**Wolf et al.*,*** [29]	Y	Y	Y	Y	Y	Y	Y	Y	Y	Y	100

Question (Q); N/A, not aplicable; Y, yes; U, unclear

Q1: Were there clear criteria for inclusion in the case series?

Q2: Was the condition measured in a standard, reliable way for all participants included in the case series?

Q3: Were valid methods used for identification of the condition for all participants included in the case series?

Q4: Did the case series have consecutive inclusion of participants?

Q5:Did the case series have complete inclusion of participants?

Q6: Was there clear reporting of the demographics of the participants in the study?

Q7: Was there reporting of clinical information of the participants?

Q8:Were the outcomes or follow up results of cases clearly reported?

Q9: Was there clear reporting of the presenting sites(s)/clinic(s) demographic information?

Q10:Was statistical analysis appropriate?

## Discussion

Odontogenic tumors (OTs) are a heterogeneous group of lesions whose pathogenesis is associated with alterations in components of signaling pathways involved in tooth formation (Wnt, BMP, FGF, Shh, and Eda) [60, 61]. The first accepted classification of OTs was published by WHO in 1971, then revised and updated in 1992, 2005, and 2017 [62], and finally, 5 years later, in 2022, thanks to advances in the technology of molecular tools used for clinical and research purposes. This new classification presents some modifications; however, based on the biological behavior and the origin of the tumor tissue, OTs are classified into benign and malignant; in turn, benign OTs are classified into epithelial, mixed (epithelial and mesenchymal), and mesenchymal, while malignant OTs are classified into carcinomas, sarcomas, and carcinosarcomas respectively [1].

Focusing on benign tumor lesions of epithelial origin, in particular AM, which are the benign counterpart of ameloblastic carcinoma, five types are currently distinguished, of which conventional AM remains the most common type, followed by unicystic and extraosseous/peripheral AM [63].

AM is a neoplasm originating from the epithelium that constitutes the enamel organ during odontogenesis [64]. It was first described in 1827 by Cusack et al. and was named “Adamantinoma” by Malassez in 1890; later, Ivy and Churchill introduced the term “Ameloblastoma” in 1930 [3, 9]. Annually, it is estimated that 0.5 cases per million population occur [65], making it one of the most frequent OTs.

A systematic review constituting the most extensive dataset (*n* = 3677) of patients with AM in different countries and regions concluded that AM presents as a painless bone swelling over the mandible, mainly in patients with a mean age of 36 years and an equal distribution by gender, very similar to the results reported in the present study. However, at the same time, there are some minor differences in the sociodemographic and clinical characteristics of AM in different countries and regions [66].

The data presented in this review are comparable with several retrospective studies published in different parts of the world. In general, it has been shown that the average age of presentation of AM ranges between the fourth and fifth decade of life [3], with a maximum age of 79 years, according to the data reported in this research. In Europe, the mean age has been reported to be 48.2 years [67]; in North America, 40.7 years [68]; in South America, 35.1 years [69]; in Africa, 31.3 years [70] and in Asia 30.35 years [71]. There is no gender predilection, although a slight male predominance has been reported, with a male-to-female ratio 3:2 [70]. Clinically, regarding their site of appearance, AM most frequently affects the posterior part of the mandible, followed by the maxilla [4, 67–72]. However, other rare extraoral presentation sites of particular interest have also been reported, such as involvement of the nasal tract (sinonasal AM), which histologically is very similar to AM of the gnathic bones, but its clinical presentation is different. It can cause partial or total obstruction of the nasal cavities and paranasal sinuses, in addition to showing typical signs of rhinorrhea and nosebleeds. Furthermore, it affects an older group of patients (59 years) [5]. On the other hand, involvement of the orbital cavities has also been reported, and the most frequent ophthalmological manifestations are decreased or loss of vision, proptosis, displacement of the eyeball, limitation of extraocular movement, diplopia, cavernous sinus syndrome, edema of the lower eyelid and ptosis [6].

The uncontrolled growth of tumors affecting the jaws can be so exaggerated that it can extend to the intracranial compartment [7], leading to severe consequences. These rare and quite dangerous locations are because the maxillary bone is very thin, fragile, and porous compared to the mandible, making it susceptible to invasion of adjacent structures without any restriction [73]. AM of the gnathic bones are usually asymptomatic and are diagnosed in advanced stages [8]. However, some authors have reported pain symptoms in the affected area [67, 74]. The presence of swelling, ulceration, malocclusion, mobility, and loss of teeth are these patients’ most frequent clinical manifestations [8]. Radiographically, the radiolucent and multilocular appearance is the most common, followed by the unilocular appearance [9, 71]. Furthermore, these lesions are characterized by displacement of adjacent teeth, root resorption, and bone expansion [67, 75]. The follicular and plexiform variants are the most frequent histological types, which can occur individually or in combination [10, 11, 67, 76].

The treatment of AM is surgical and can be performed using a conservative approach, which includes enucleation, curettage/curettage, and/or marsupialization, as well as a radical approach that provides for marginal and segmental resection, hemimandibulectomy and/or hemimaxylectomy, depending on the extent and severity of the lesion. In fact, according to scientific evidence, treatment strategy is the main factor influencing recurrence rates and the risk of developing postoperative complications [13–16]. A study that analyzed 158 cases showed that the risk of recurrence of AM treated by enucleation was 4.62 times higher than that of AM treated radically [77]. Very similarly, another study reported a higher recurrence rate in patients undergoing bone curettage and enucleation compared to patients treated radically; however, the latter presented a more significant number of complications (facial asymmetry, temporal paresthesia of the inferior alveolar nerve, infection, and swelling), aesthetic and functional deficiencies that could compromise the quality of life of patients [78]. This agrees with another study comparing surgically treated AM patients’ pre- and post-operative quality of life. The authors found that quality of life decreased immediately after surgery, then gradually improved and exceeded preoperative values 6 months after the surgical procedure [15]. Therefore, surgical excision with wide safety margins (1.5 to 2 cm) is the mainstay for treating conventional AM since conservative approaches show high recurrence rates.

Secondary defects of the oral and maxillofacial region that occur as a consequence of radical surgical procedures can often lead to severe aesthetic and functional alterations, requiring complex reconstructive techniques with the primary purpose of achieving oral rehabilitation, that is, restoring the patient’s symmetry and functionality, as close as possible to their premorbid state and, therefore, improve their quality of life [79].

Scientific evidence has suggested that the vascularized fibula free-flap is the most widely used autogenous bone graft for the reconstruction of orofacial anatomical defects as a consequence of surgery [80], which is consistent with the results of our study. This flap type provides adequate bone length, a long vascular pedicle that easily adapts to the mandible, and a bicortical architecture, which increases the primary fixation of dental implants [81]. In this sense, high success rates (91%) have been reported through the use of this type of graft [82], the most frequent complications being the presence of infections and wound dehiscence, loss of skin graft in the donor site, complete flap loss and percutaneous fistulas [83]. Furthermore, oral health-related quality of life has been shown to improve markedly in these patients [84]. Finally, it is expected that, with new technologies, such as virtual surgical planning with 3D models and cone beam computed tomography, more precise reconstructions can be made, reducing the risk of postoperative complications and favoring subsequent implant-supported prosthetic rehabilitation.

Of the studies evaluated, none reported recurrences of the tumor lesion after dental implant placement, demonstrating the surgical treatment’s high effectiveness. Furthermore, the survival rate of dental implants was 98.1%. In general, survival/success rates of 83.9% [85], 97% [22], and 98.8% [86] have been reported, demonstrating that oral rehabilitation with dental implants inserted in free flaps for reconstruction maxillofacial surgery after ablative surgery can be considered a safe treatment modality with successful results.

On the other hand, in cancer, it has been shown that patients mainly benefit from the primary placement of dental implants for prosthetic rehabilitation with a 5-year survival rate of 92.8% compared to secondary placement (86, 4%) [19], as well as, for those patients who have not received radiotherapy compared to previously irradiated sites [21]. Wuster et al. [87] reported high survival rates in patients who had undergone surgery for a head and neck tumor. A survival rate of 99.1% after 3 years and 93.1% after 5 years was reported in patients without vestibuloplasty, compared to a survival and implant success rate of 100% after 5 years in patients with vestibuloplasty, which improved considerably. During the observation period, six implants were lost. Therefore, the authors suggest that vestibuloplasty should always be considered and applied if the anatomical conditions require it to achieve high implant success rates in patients with head and neck tumors.

A high survival rate of dental implants has been demonstrated in patients with systemic autoimmune diseases with repercussions in the oral cavity. In patients with lichen planus, and after a follow-up period of 44,6 months, the survival rate of dental implants was 98,3%. In patients with epidermolysis bullosa it was 98,7% at 32,6 months. For patients with Sjögren’s syndrome it was 94,2% after 45,2 months. In patients with systemic sclerosis it was 97,7% after 37,5 months. In patients with pemphigus, and systemic lupus erythematosus it was 100% after 24 months [88] Thus, it appears that the autoimmune status had no discernible impact on the survival rate of dental implants, however, a comprehensive risk assessment is recommended before starting implant therapy [89].

Stable anchorage of fixed and/or removable implant-supported dental prostheses represents successful clinical treatment approaches in partially edentulous patients [90]. To achieve an adequate osseointegration process, implants should be immersed in the bone without placing any type of load for 3 to 4 months (early loading) up to 6 to 8 months (conventional/late loading) [91]. In the present study, the conventional approach was the most used by surgeons, and although the immediate loading (placement of the provisional/definitive on the same day of surgery) of dental implants has benefited by shortening the treatment period. Available randomized clinical trials suggest that this protocol is associated with a higher incidence of implant failure [92]. To allow immediate placement or provisionalization, good initial stability of the implant is required (> 35 N/cm2), and an implant stability coefficient value > 60 will allow a good prognosis for the patient. However, good primary stability is not always possible, and some factors such as local anatomy, bone density, implant milling protocol, and macro-design may influence it [93–95]. Finally, hybrid prosthetic restorations represent an excellent option for reconstructing alveolar ridges with moderate to severe resorption. They correspond to a screw-retained structure with cemented crowns fabricated of different materials (porcelain, lithium disilicate, zirconia). These restorations splint the implants together, provide adequate resistance, and meet aesthetic demands [56, 96–101].

## Conclusions

Based on the findings found in this review, the following conclusions can be drawn:

1. Conventional AM follicular histological variant was the most common type of tumor lesion.
2. The treatment of choice was surgical resection of the tumor using segmental mandibulectomy.
3. The fibula-free flap was used most frequently for the reconstruction of the orofacial defect, and on average, the time elapsed after the maxillofacial reconstruction for the placement of dental implants was 23 months, equivalent to 1.9 years.
4. An implant survival/success rate of 98.1% was reported. Furthermore, most of them were loaded conventionally.
5. Hybrid implant-supported fixed dental prostheses were the most used by prosthodontists.

## Data Availability

The data supporting this study’s findings are available from the corresponding author upon reasonable request.

## Abbreviations

AM: Ameloblastoma
OTs: Odontogenic tumors
WHO: World Health Organization
Wnt: Wnt/B-catenin signaling
BMP: Bone morphogenic protein
FGM: Fibroblast growth factor
Shh: Sonic hedgehog
Eda: Ectodysplasin A
